# Clinical characteristics of magnetic foreign body misingestion in children

**DOI:** 10.1038/s41598-021-96595-y

**Published:** 2021-09-21

**Authors:** Yu Kun Huang, Shao Xian Hong, I. Hsin Tai, Kai Sheng Hsieh

**Affiliations:** 1 Department of Critical Care Medicine, Children’s Hospital of Fudan University Xiamen Branch, Xiamen, 361006 Fujian China; 2 Department of Pediatric Emergency & Cardiology, China Medical University Hospital, China Medical University, Taichung, 40447 Taiwan; 3 Department of Medicine, College of Medicine, China Medical University, Taichung, 40447 Taiwan; 4 Department of Pediatrics, Taipei Medical University-Shuang Ho Hospital, New Taipei City, 23561 Taiwan

**Keywords:** Health care, Stomach

## Abstract

Magnetic foreign body misingestion (MFBM) is now occurring more frequently. It may cause remarkable mortality and morbidity in children. A retrospective analysis of the clinical data of children admitted to Xiamen Children’s Hospital between March 2017 and July 2020 due to accidental MFBM. A total of 14 children who had MFBM were collected, the proportion between urban and rural areas was 8:6, and the ratio of male to female was 6:1. The age ranged from 1.2 to 8.9 years (median 4.6 years). The number of magnetic foreign bodies ingested by mistake is 1 to 17 (average 6.5). Magnetic foreign objects are divided into magnets (3 cases) + magnetic beads (11 cases). About 40% (5/14) of this patient series showed no available misingestion history. Management includes: 4 cases of open surgery (including 1 case of laparoscopic transfer to operation), 3 cases of laparoscopic surgery, 2 cases of gastroscopy, 5 cases of conservative treatment of foreign bodies discharged through the anus. Of the 7 surgical cases, 6 cases presented with intestinal obstruction and intestinal perforation (at least 1 intestinal perforation and at most 5). Abdominal sonography has limitations in the detection of magnetic foreign bodies in the digestive tract. The proportion of laparoscopic surgery in the 7 surgical cases is nearly half. All surgical cases recovered smoothly after treatment. Our experience shows that MFBM is a big issue for the small children! The early symptoms of MFBM are often atypical especially among young children and MFBM may lead to severe adverse events. We proposed a management strategy for MFBM in children. We advise pediatricians/emergency physicians, parents/children’s guardians and society should raise the collaborated alertness of MFBM. Global awareness of risk prevention of magnetic material accidental ingestion cannot be overemphasized.

## Introduction

Foreign bodies in the digestive tract of children have become one of the important accidental injuries in children. The first case of foreign bodies in the digestive tract of children was reported in 1692^1^. It was the 4-year-old Prince Brandenburg who swallowed a leather shoe fastener. Since then, similar situations are not uncommon, and the consequences and complications caused by this are quite different. It mainly depends on the shape, nature, and quantity of the foreign body. Its outcomes ranged from the spontaneous discharge, intestinal perforation, obstruction, bleeding, and even sepsis, septic shock, etc^2,3^. Some of the complications are life-threatening. To clarify the clinical status of MFBM in children, we, therefore, conducted this study.

## Materials and methods

We respectively retrieved and reviewed the clinical data of all children admitted to Xiamen children’s Hospital (XMCH), including age, gender, clinical manifestations, medical history and signs, imaging examinations, treatment, and outcome, etc.

The diagnostic criteria for magnetic foreign bodies were based on the history of accidental swallowing of magnetic foreign bodies, clinical manifestations, abdominal signs, and abdominal imaging examination (abdominal X-ray and CT examination). The relevant data were analyzed and presented. The study was approved by the institutional review board of Xiamen children’s Hospital. The authors have identified the institutional and/or licensing committee approving the experiments, including any relevant details; confirming that all experiments were performed in accordance with following relevant guidelines and regulations. Informed consent was waived by the institutional board of Xiamen children’s hospital as a retrospective study is performed.

## Result

### General information

Between March 2017 and July 2020, 14 cases of children with MFBM were admitted to Xiamen Children’s Hospital, including 12 males and 2 females. The ratio of urban to rural areas is 8:6, and the ratio of males to females is 6:1. The age span is between 1.2 years old and 8.9 years old, and the median age is 4.6 years old. The number of ingested magnetic foreign bodies ranged from 1 to 17, with an average of 6.5. The properties of magnetic foreign bodies are divided into 3 cases of magnets and 11 cases of magnetic beads.

### Clinical symptoms

The main manifestations of 14 children were abdominal pain, bloating, vomiting, fever, etc. Among them, 6 cases were complicated by intestinal obstruction and intestinal perforation (1–5 locations). Some of them had no clinical symptoms due to the short time of ingesting foreign bodies. Of the 14 cases, 9 cases were able to provide a history of ingesting foreign bodies at the time of medical visit, and the other 5 children and their families could not provide a history of ingesting foreign bodies. The clinical characteristics of these children without a history of MFBM are shown in Tables 1 and 2 showed clinical characteristics of these children with a traceable history of MFBM. The cases in group 1 had a complicated clinical course and longer hospital stay (median 13 days, range 10–22 days) than those in group 2 (median 3 days, range 2–12 days) as shown in Table 3.

**Table 1:** Clinical characteristics of 5 children with unwitnessed/non-self-reported magnetic foreign body misingestion in children.

No	Age, year	Gender	Interval between time of misingestion to clinical visit	Number of FB/initial recognition site	Symptom(s)	Complication	Treatment	Hospital stay, days
1	8.9	F	> l day (unwitnessed, patient had visited other hospital with negative abdomen sonography 1 day before admission)	2 Mags/XMCH	Abdominal pain, vomiting	Obstruction, 4 perforation sites	Lap-tomy	10
4	3.9	M	> 2 days (unwitnessed, had visited 2 other hospitals and KUB at 2nd hospital revealed magnets)	14 MagBs/OH	Abdominal pain, vomiting, fever	Obstruction, 2 perforation sites	Lap-tomy	10
6	5	M	> 14 h (unwitnessed)	17 MagBs/XMCH	Abdominal pain, vomiting	Obstruction, 1 perforation sites	Lap-sco	10
7	1.2	M	> 2 days (unwitnessed)	7 Magils/XMCH	Vomiting, bloating, fever, crying	Obstruction, 5 perforation sites Sepsis	Lap-sco	22
8	1.4	F	> 6 days (unwitnessed, patient had visited 2 hospitals in 6 days and KUB was not done until 6th day of onset of symptoms)	3 MagRs /OH	Abdominal pain, vomiting	Obstruction, 2 perforation sites	Lap-sco	13

*FB* foreign bodies, *Mag* magnet, *MagB* magnet bead, *XMCH* Xiamen Children’s Hospital, *OH* another hospital, *Lap-tomy* laparotomy, *Lap-sco* laparoscopy.

**Table 2:** Clinical characteristics of 9 children with witnessed/self-reported magnetic foreign body misingestion.

No	Age, year	Gender	Interval between time of misingestion to clinical visit	Number of FB/initial recognition site	Symptom(s)	Complication	Treatment	Hospital stay, days
2	8.5	M	5 h (witnessed, visited other hospital with only supportive care without KUB)	2 Mags/XMCH	Abdominal pain, vomiting	Obstruction, 1 perforation sites	Lap-tomy	12
3	3.3	M	2 h (witnessed and sent to XMCH directly)	2 Mags/XMCH	No	No	PES-rc	3
5	5	M	5 h (witnessed)	12 MagBs/XMCH	Abdominal pain	No	Lap-tomy	9
9	4.5	M	6 h (witnessed, patient visited other hospital and KUB confirmed diagnosis)	4 MagBs/OH	No	No	Supportive care	2
10	6.4	M	2 hours (witnessed)	8 MagBs/XMCH	No	No	Supportive care	2
11	2.8	M	8 (witnessed, patient visited other hospital and KUB confirmed diagnosis)	7 MagBs/OH	Vomiting	No	PES-re	4
12	5.2	M	2 h (witnessed, patient visited other hospital and KUB confirmed diagnosis)	1 MagBs/OH	No	No	Supportive care	2
13	3	M	6 h (witnessed, patient visited other hospital and KUB revealed magnets)	11 MagBs/OH	No	No	Supportive care	3
14	4.7	M	1 h (witnessed)	3 MagBs/OH	Na	No	Supportive care	2

*PES-re* panendoscopy retrieval.

**Table 3:** Comparison of unwitnessed/non-self-reported magnetic foreign body misingestion (Group 1) and witnessed/self-reported magnetic foreign body misingestion (Group 2) in children.

Group	Event witnessed/self-reported	Interval between misingestion and GI symptoms	Initial KUB at 1st medical visit site	GI symptoms	Gastroscopic retrieval	Discharged with stool	Operation	Hospital stay, days
1 (N = 5)	No	Unknown	3/5	5/5	0/5	0/5	5/5 (3 Lap-sco, 2 Lap-tomy)	10–22 days (median: 13 days)
2 (N = 9)	Yes	1–8 h	3/9	4/9	2/9	5/9	2/9 (2 Lap-tomy)	2–12 days (median: 3 days)

### Image analysis

**Figure 1:**
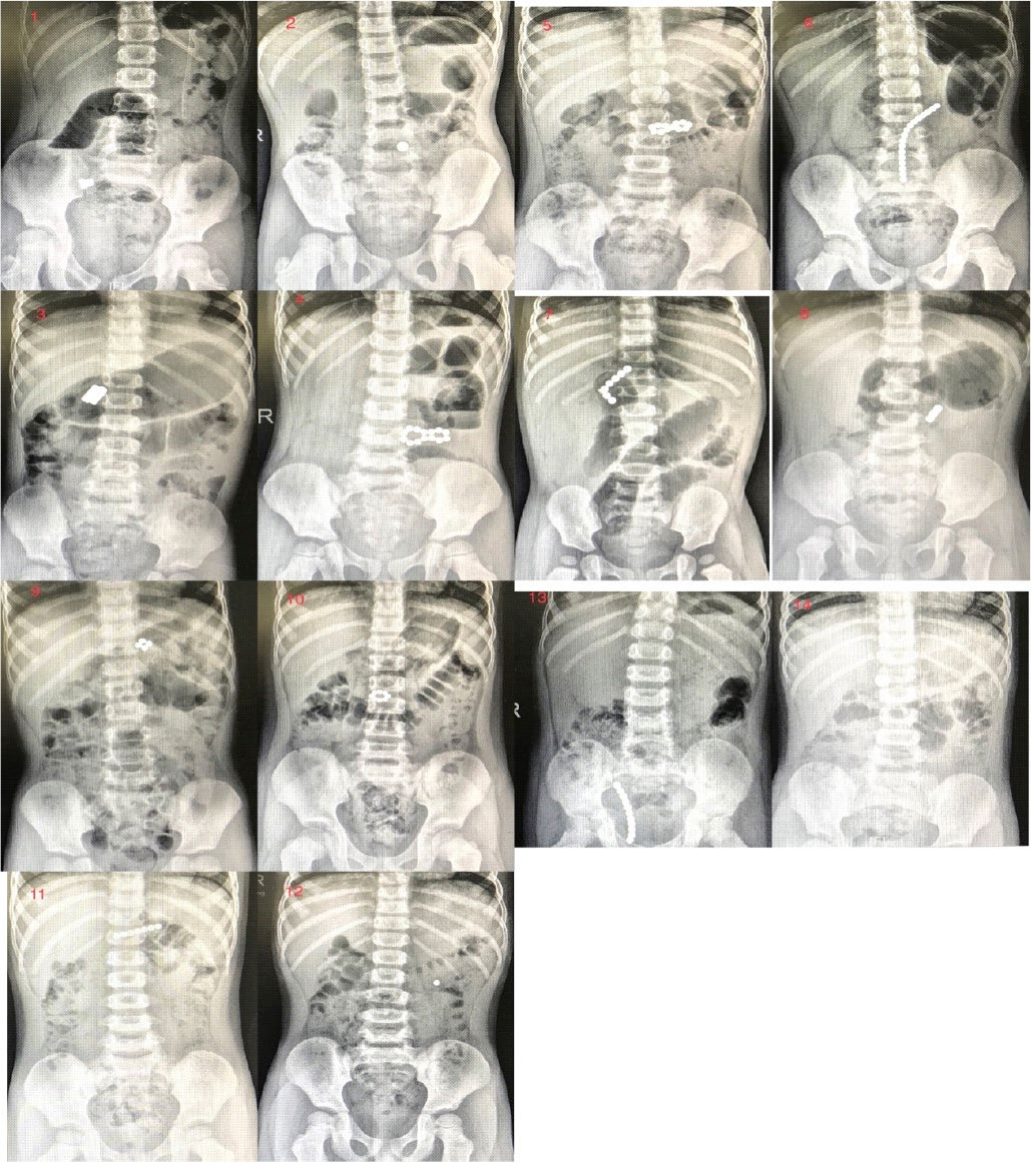
Abdominal X-rays of the 14 children with different number and different locations of the misingested magnets/magnetic beads (please refer to Tables 1, 2 in detail).

1. The abdominal X-rays of all 14 children (Fig. 1) clearly showed foreign bodies in the digestive tract. In cases No. 5 and 9 it appeared that the location of the magnetic foreign body was in the stomach. However, the subsequent gastroscope showed there was no foreign body in the stomach. The misingested magnetic foreign bodies showed various shapes of stacks of magnets.
2. The abdominal sonography before surgery did not indicate the presence of foreign bodies in the digestive tract in cases No. 1, No. 2, 5, 7, and 8. Neither had it showed magnetic foreign bodies in case 1 who had sought medical help in another hospital before arrival at XMCH.

### Management and outcome

Gastroscopy was performed on 2 cases (case 5 and case 9) but failed to show the magnets in the stomach. In case 5, laparotomy was performed to retrieve the magnets. While in case 9, conservative care was made and spontaneous discharge occurred 3 days later. Seven of the 14 cases underwent surgical treatment to remove foreign bodies (including 4 cases of open surgery and 3 cases of laparoscopic surgery); of the 7 cases underwent an operation, 6 cases had a complicated intestinal obstruction and intestinal perforation (at least 1 intestinal perforation, at most 5 Place). Two of the 14 cases showed that the location of the foreign body was in the stomach by the abdominal X-ray, and the gastroscopy did not show the foreign body in the stomach. The proportion of laparoscopy in the 7 surgical cases is nearly half, including case 2 who initially had laparoscopy but subsequently switched to open laparotomy, suggesting that laparoscopic exploration can be used as the preferred treatment for intestinal perforation and/or intestinal obstruction caused by magnetic foreign bodies in the digestive tract. All surgical cases recovered smoothly after treatment, and no complications occurred during the longest follow-up period of 6 months.

## Discussion

Foreign bodies in the digestive tract are not uncommon in children. In recent years, related reports have gradually increased, especially the reports of magnetic foreign bodies. This is related to the wide application of magnetic materials in children's toys. Among the 14 cases in this group, 12 were boys and 2 were girls. The ratio of urban to rural areas was 8:6. The age span was between 1.2 and 8.9 with a median of 4.6. The proportion of boys is much higher than that of girls, accounting for 85.7%, which is related to boys’ active and naughty nature; in terms of age proportions, there are 3 infants, 9 preschoolers, 2 schooler children, indicating 64% preschoolers. This is probably related to the strong curiosity of preschool children, the lack of relevant safety knowledge, and the weak sense of alertness of parents/guardians.

Compared with other foreign bodies, misingestion of magnetic foreign bodies in the digestive tract is unique and needs to be paid attention to. In contrast to other kinds of foreign bodies, the magnetic foreign bodies (such as magnets, magnetic beads) are easily attracted to each other (if the misingested number is 2 or more) in the digestive tract, as reflected in the abdominal X-rays of these patients. The aggregation of magnets may thus cause the compression of the luminal wall of the gastrointestinal tract resulting in ischemic necrosis, which can cause single or multiple intestinal perforation, intestinal obstruction, peritonitis, intussusception, and hemorrhage^3–6^. In severe cases, it can lead to sepsis, septic shock and life-threatening^3^. Of the 14 cases in this series, 2 cases ingested a single magnetic foreign body while the remaining 12 cases misingested multiple magnetic foreign bodies, which accounted for as high as 85.7% among the 14 children with MFBM. This is a higher proportion compared to the 67% of children who mis-ingested multiple magnets or additional metal foreign bodies reported by Sola et al.^7^. Therefore, in view of the occurrence rate of multiple magnetic foreign bodies and its possible serious complication, the proportion of surgical operations is higher than other kind of foreign bodies. Compared with the damage caused by multiple magnetic foreign bodies, a single magnetic foreign body can often be expelled along with feces by itself, and the outcome is relatively better.

In this group of 14 children, 9 cases clearly provided medical history at the time of medical visit, 5 (35.7%) cases failed to provide adequate medical history, and all these 5 cases had an intestinal obstruction and intestinal perforation (1–5 locations). One case was even complicated with sepsis. Thus, the clinical medical history often is unclear for foreign body misingestion in children. Because of the possible serious complications for children with MFBM, it is therefore particularly important that for children with unexplained abdominal symptoms, foreign body misingestion, particularly magnetic foreign body misingestion should be considered because the children who misingested foreign body often could not express the fact and abdominal plain X-ray should be taken accordingly.

Abdominal sonography, although with many advantages such as non-radiation, high portability, and non-invasiveness etc., may not be helpful in children with MBFM. In our series, 4 of them underwent abdominal sonography in our hospital and another one had abdominal sonography in another hospital before admission to our hospital. In all 5 cases the abdominal sonography failed to obtain positive information. Therefore, until more experience is accumulated, abdominal sonography is not considered for diagnosis of MFBM in children.

There were 2 children who underwent abdominal X-ray examination which indicated that the foreign body was in the stomach, but the emergency gastroscopy results showed that there was no foreign body in the stomach. This might be due to time lag between the abdominal X-ray and gastroscopy or due to an orthogonal plane of abdominal X-ray that may be needed to localize the exact location of the magnetic foreign body. In another case, the abdominal X-ray (anterior position) showed the illusion of “1 magnet” at the time of presentation, but the lateral radiograph showed “2 magnets”, again suggesting that at least orthogonal X-ray examination should be performed for cases with misingestion of magnetic or metal foreign bodies in the digestive tract. Kircher et al.^8^ have reported related similar situations.

There is currently no consensus on how to deal with a single misingested magnetic foreign body. At present, Clinicians generally agree that conservative observation and dynamic follow-up of abdominal X-rays. Most single magnetic foreign bodies can be expelled with feces by themselves. Two of the 14 cases in this series misingested single magnetic foreign bodies, which were all conservatively managed. The magnetic foreign bodies were eventually discharged through the anus on their own without complications such as intestinal perforation or obstruction. Scholar Sun Jun et al.^9,10^ suggested that children who accidentally ingested multiple magnets should be actively treated with surgery, regardless of their clinical manifestations. Among the 14 cases in this series, multiple magnetic foreign bodies accounted for 12 cases, but 7 cases were removed by surgery and 2 cases were removed by gastroscope. In this group of cases, 6 out of 7 undergoing surgery showed complicated intestinal perforation (the perforation sites were up to 5), but in all cases of intestinal perforation, the abdominal X-ray did not show obvious free gas under the diaphragm. However, it was confirmed that the gastrointestinal perforation was present, all with multiple sites of perforation, during the operation. This is probably due to the surrounding effect of adhesive tissue. Therefore, it should be noted that the absence of free gas does not rule out the absence of intestinal perforation. Three cases were managed conservatively.

Intestinal perforation caused by multiple magnetic foreign bodies is often located in multiple locations, scattered and hidden. Therefore, regardless of the choice of laparotomy or laparoscopic exploration, the entire digestive tract (from the stomach to rectum) should be explored to ensure that no digestion is missed Tract perforation and no missing magnetic foreign body, if necessary, X-ray should be rechecked during the operation to avoid undetected magnet(s) ; in this group of cases, there are 3 cases of multiple foreign bodies (the number of foreign bodies is 4, 8, 11) foreign bodies were expelled spontaneously after conservative management. Among them, the child who misingested 11 magnetic foreign bodies expelled them in 13 days.

In addition, a total of 4 patients had clinical followed up, the follow-up time was 1 month, 1 month, 5 months, and 6 months. The remaining cases were not followed up due to reasons such as residence. All these cases had all paroxysmal abdominal pain, the degree and frequency of abdominal pain varied; there was no accompanied vomiting, diarrhea, and intestinal obstruction. The abdominal pain was relieved spontaneously. The follow-up radiologic imaging all showed no obvious abnormal lesions.

Based on this series, we thus suggest a protocol in handling mis-ingested multiple magnetic foreign bodies is as follows: 1. For children with multiple MFBM within 4 h and confirmed their presence in the stomach region on the orthogonal abdominal X-ray, an emergent gastroscopy should be done immediately/as soon as possible to decrease the time interval for gastroscopy. 2. For children who misingest multiple magnetic foreign bodies or for a long time (more than 4 h) or accompanied by abdominal pain, vomiting, or bloating, fever, etc., we recommend emergency surgery. The indications for surgery cannot be judged solely on the results of abdominal X-ray examination and other signs of intestinal perforation, such as free air under the diaphragm. Regarding the choice of surgical methods, laparotomy or laparoscopic exploration can be selected according to the situation of the child and the surgeon's preference. Laparoscopy may be attempted first. 3. For children who misingested multiple magnetic foreign bodies, but the foreign body has passed through the stomach and duodenum and cannot be removed by the gastroscope, present no abdominal pain or vomiting, no abdominal distension, no fever and no other symptoms, and show normal stools, and abdominal X-rays did not indicate intestinal perforation, intestinal obstruction, intestinal motility changes, can continue to observe and follow-up^11^.

In summary, pediatricians/emergency physicians should be vigilant about the hazards caused by misingestion of magnetic foreign bodies in children. They should make great effort to make differential diagnosis among children with gastrointestinal symptoms and to recognize magnetic foreign bodies as early as possible, actively manage them, reduce complications, and strengthen publicity. For magnetic foreign bodies in the digestive tract, abdominal X-ray examination has a higher diagnostic value than ultrasound, but additional orthogonal plane may be attempted to better localize the location of the magnetic foreign bodies in the abdomen. Also, the management planning should be judged according to the number and size of the foreign body, the number of misingestion magnets and the condition of the children. For children who misingested multiple magnetic foreign bodies, or mis-ingested it multiple times, and who presented with intestinal obstruction or pneumoperitoneum, active surgical operations are required. For management, laparoscopy may be the first choice.

Our experience shows we encountered 14 cases of MFBM in about 2.5 years. To the best of our knowledge, this is the largest series of MFBM in a single center in such a short period. Thus MFBM is really a big issue for small children. In view of the harm caused by magnetic foreign bodies in the digestive tract, we hereby call for: (1) Children’s parents/guardians and the whole society should be repeatedly educated through multiple channels to let them alert the seriousness of the accidental MFBI, strengthen guardianship, take responsibility on-site, and reduce the incidents of MFBM; (2) currently, Many countries probably have various existing laws/regulations on toy production and quality, but in fact there are deficiencies to earnestly inform toy risks and related conditions and to implement more effective measures to prevent children, especially small children from being exposed to the risk of MFBM, It is a serious big global issue on the further focus of relevant laws and regulations to strictly supervise small children’s magnetic toys and eliminate them from the source; the family and society’s awareness of the risk of accidental ingestion of magnetic materials should always be emphasized.
